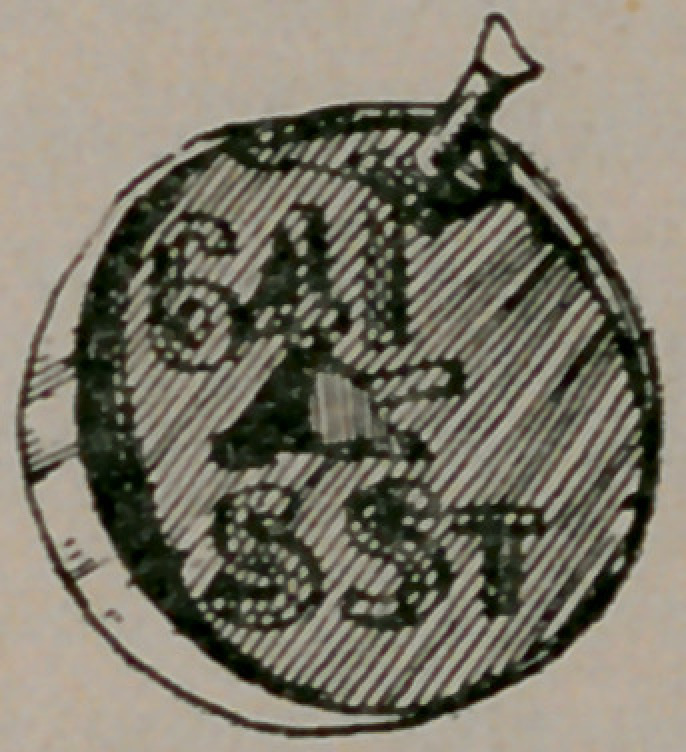# Answers to Correspondents

**Published:** 1899-11

**Authors:** 


### CORRESPONDENCE.

---

### ANSWERS TO CORRESPONDENTS.

MIDDLE OF THE ROAD, GA.—Mr. Editor: Valuing your ability to help all who are in doubt, please advise me about this case: I have a female patient 90 years old. She has lost flesh and cannot walk very erectly. She also has some difficulty in masticating her food. She also sleeps in her chair in daytime.

INN THEE DARKE, M.D.

ANSWER: This is easy. Doctor, your patient is suffering from marasticity and incipient rickets. Give her the mixture known as cornis-vin-morrh-calc, tie a board to her back-bone and have her buy a family faradic battery. The remedy will restore flesh, the board will hold her up and the battery can be used to wake her up. If she can't chew get her a sausage-grinder.—ED.

---

CAN'T GET AWAY, MO.—Mr. Editor: My horse has a boring pain in his abdomen. Is it appendicitis?

SAMPEL COPPY, M.D.

ANSWER: It is botts. Give your patient a minnow to eat the bott, or put on plaster of paris belly-band to keep it from boring out.—ED.

FAYETTEVILLE, GA., 1899.

*Atlanta Journal-Record of Medicine:*

On June 21st, about 3 P.M., while swinging in a hammock, lying on her back, a little girl three and a half years old, and very delicate, the child of my partner, Mr. J. R. Murphy, swallowed the enclosed (a car seal), which she happened to be playing with and had in her mouth.

Her mother was in another room and heard her choke; ran to her, but she said she had swallowed it, and told her mother what it was. The lady sent to the store for her husband, who appealed to me to know what to do. I told him to do nothing, as I feared to give an emetic, for, it being lead, I knew it would at once sink to the bottom of stomach and could not be brought back without danger. I feared a purgative, knowing the jagged wire attached would endanger a rupture if forced. So we decided to trust to nature.

On the 23d, about the same time in the evening, it passed from her, being partially embedded, but not solidly so, as it separated from the other substance and rolled to one side when exposed to the air. My views are that we often endanger the lives of our patients by giving way to a desire to do something, feeling that we ought at least to satisfy the family by giving something and leaving the impression that if we hadn't been called the patient might have died. We should be honest always with them, and in such cases simply assure them that nature will do all that can be done, and wait until it has time to act, and then, if a necessity arises, dose them.

This little girl complained of pains in her bowels, but had been complaining of the same before, and we don't think the accident caused a single pain or had any effect whatever on her, as it has been five days since it passed from her and she is all right.

Yours truly, J. E. TUCKER, M.D.

DEPARTMENT OF THE INTERIOR, CENSUS OFFICE,
WASHINGTON, D. C., September 15, 1899.

*To the Editor Journal-Record of Medicine:*

DEAR SIR:—Appreciating the important aid your influential journal can give the Census Office in its present effort to secure the adoption of a uniform certificate for the return of deaths and looking toward the establishment of a national system of mortality registration, the Director of the Census has instructed me to call your attention to the circular (7-207) enclosed and to prepare for you the accompanying condensed statement of facts (7-234) contained in the former which is intended for publication, if you feel warranted in so doing.

The circular fully details the steps that have been taken in this direction by the Census Office and shows that nearly all of the registration officers, with whom correspondence has been had, have courteously and promptly given the assurance desired. They promise generally to amend or recast their local death-certificate so as to embrace all the Census requirements and to put the improved form in use for the circular year 1900, which is the period selected for the collection of mortality returns.

Cooperation has thus been practically secured, which is all that can be accomplished by the office in this direction, but this in itself does not insure the satisfactory return of the information required for the Census statistics. Something further is necessary.

It is apparent that upon the medical fraternity of the United States, more than upon any other agency involved, depends the ultimate success of the project, as the prompt and correct return, upon the new death certificates, of the facts required by Congress for the Census is the chief essential. The director believes that their active cooperation and interest in a reform that promises such results to the cause of science may be relied upon if the journals that so thoroughly disseminate medical knowledge bring it to the attention of their readers.

Trusting that your journal will do all it can to enlist its clientele of physicians in promoting national uniformity and directing your especial attention to the concluding paragraph of the circular, I have the honor to be

Very respectfully,
W. A. KING, Chief Statistician.

## Figures and Tables

**Figure f1:**